# Genetic characterization of MDR genomic elements carrying two *aac(6′)*-*aph(2″)* genes in feline-derived clinical *Enterococcus faecalis* isolate

**DOI:** 10.3389/fmicb.2023.1191837

**Published:** 2023-07-27

**Authors:** Xue-Song Li, Yu Qi, Peng-hui Li, Jun-ze Xue, Xuan-yu Li, Inam Muhammad, Ya-zhuo Li, Dao-mi Zhu, Ying Ma, Ling-Cong Kong, Hong-Xia Ma

**Affiliations:** ^1^Department of Veterinary Medicine, College of Animal Science and Technology, Jilin Agricultural University, Changchun, China; ^2^The Key Laboratory of New Veterinary Drug Research and Development of Jilin Province, Jilin Agricultural University, Changchun, China; ^3^Department of Zoology, Shaheed Benazir Bhutto University, Sheringal, Pakistan; ^4^Liaoyuan Animal Disease Prevention and Control Center, Liaoyuan, China; ^5^The Engineering Research Center of Bioreactor and Drug Development, Ministry of Education, Jilin Agricultural University, Changchun, China

**Keywords:** *Enterococcus faecalis*, multidrug-resistant, whole genome sequencing, mobile genetic elements, transposon

## Abstract

Multidrug-resistant *Enterococcus faecalis* (*E. faecalis*) often cause intestinal infections in cats. The aim of this study was to investigate a multidrug-resistant *E. faecalis* isolate for plasmidic and chromosomal antimicrobial resistance and their genetic environment. *E. faecalis* strain ESC1 was obtained from the feces of a cat. Antimicrobial susceptibility testing was carried out using the broth microdilution method. Conjugation experiments were performed using *Escherichia coli* and *Staphylococcus aureus* as receptors. Complete sequences of chromosomal DNA and plasmids were generated by whole genome sequencing (WGS) and bioinformatics analysis for the presence of drug resistance genes and mobile elements. Multidrug-resistant *E. faecalis* ESC1 contained a chromosome and three plasmids. The amino acid at position 80 of the *parC* gene on the chromosome was mutated from serine to isoleucine, and hence the amino acid mutation at this site led to the resistance of ESC1 strain to fluoroquinolones. Eleven antibiotic resistance genes were located on two plasmids. We identified a novel composite transposon carrying two aminoglycoside resistance genes *aac(6′)*-*aph(2″)*. This study reported the coexistence of a novel 5.4 kb composite transposon and a resistance plasmid with multiple homologous recombination in an isolate of *E. faecalis* ESC1. This data provides a basis for understanding the genomic signature and antimicrobial resistance mechanisms of this pathogen.

## 1. Introduction

Humans have begun mass production and usage of antibiotics since long for the treatment of infectious diseases due to which many bacteria have evolved resistance to a variety of antibiotics (D’Costa et al., 2011). An important driver of the persistent evolution of existing mechanisms of drug resistance could be the endless competition for resources among microorganisms, including the natural production of secondary metabolites by microorganism which are used against many antibiotics today (Allen et al., 2010). The introduction of relatively new antibiotics as clinical drugs has radically altered the conditions for the evolution and spread of drug resistance by exerting unprecedented selection pressure on members of the microbial community, which has not only contaminated the humans and livestock but the environment as well (Larsson and Flach, 2022). This selective pressure has promoted the mobilization and horizontal transfer of a large number of antibiotic resistance genes (ARGs) to many bacterial species, especially to pathogenic bacteria (Alcock et al., 2020). The end result of this cumulative evolutionary event has been known to increase difficulty in preventing and treating bacterial infections. Bacteria and resistance genes not only transmit amongst species and environments but could lead to transboundary diseases as well, therefore, one health concept is the only way for understanding and recognizing the links between human, animal, and environmental microflora and addressing this global health challenge (Mackenzie and Jeggo, 2019; Buschhardt et al., 2021).

Recently, COVID-19 is considered as one the common, prominent and alarming illness among infectious diseases which has increased community’s awareness regarding public health issues and they have realized that zoonotic diseases could be the similar threats for creating pandemic situations (Fritz et al., 2021). The companion animals that live with humans day and night can be the source of zoonotic diseases and pose a potential threat to human health (Powell et al., 2022). There is a large number of microflora in the intestinal flora of companion animals (Ganz et al., 2022), and among them, *Enterococcus faecalis* (*E. faecalis*) is a pathogenic anaerobic gram-positive *Enterococcus* that is an early colonizer of the intestine in humans and animals (Chong et al., 2017; Willett et al., 2021). Yuan et al. (2023) monitored *E. faecalis* in the intestines of domestic cats in Northeast China and found that the isolation rate of *E. faecalis* in fecal samples from domestic cats was 76.67%. At the same time, the isolated strains were found to be resistant to tetracycline and erythromycin (Yuan et al., 2023). It is also a conditional pathogen that has been recognized as an important cause of nosocomial infections increasingly. Similarly, it can cause related infections, including bacteremia, bacterial endocarditis, amyloid arthropathy, diarrhea and gastrointestinal inflammation (Cauwerts et al., 2007; Gregersen et al., 2010; Fan et al., 2021; Guo et al., 2022). Recently, multidrug-resistant *E. faecalis* has been frequently reported (Kang et al., 2021; Stȩpień-Pyśniak et al., 2021; Yoon and Lee, 2021; Shan et al., 2022). The emergence of antibiotic-resistant strains has brought challenges to clinical practice and potential threat to public health (Anderson et al., 2018).

Antibiotic resistance in *E. faecalis* mediated by mobile genetic elements has been frequently reported. Tn*916* is a conjugative transposon originally found in *E. faecalis* DS16. It confers resistance to tetracycline via *tet*(M) (Franke and Clewell, 1981; Flannagan et al., 1994; Browne et al., 2015). Tn*1331* is composed of a Tn*3* transposon (carrying *bla*TEM-1 gene) and two gene cassettes carrying three antibiotic resistance genes, namely *aac(6′)-Ib*, *ant(3″)-Ia* and *bla*OXA-9 genes, conferring resistance to kanamycin, streptomycin and beta-lactams, respectively (Alavi et al., 2011). Tn*6674* is a 12.9-kb Tn*554*-related transposon, which was found in a porcine *E. faecalis* strain E1731, it contains four resistance genes [*spc*, *erm*(A), *fex*A and *optr*A] conferring resistance to spectinomycin, macrolides, phenicols and oxazolidinone, respectively (Li et al., 2019). Tn*6248* carries *tet*(M), *tet*(L) and *cat*A genes, conferring tetracycline and chloramphenicol resistance (Gawryszewska et al., 2017). In addition, enterococci with high-level gentamicin resistance (HLGR) have also been reported in foods of animal origin (Choi and Woo, 2013; Liu et al., 2013). A study by Zhang et al. (2018) indicated that a composite transposon carrying the *aac(6′)*-*aph(2″)* gene mediated high levels of resistance to aminoglycoside antibiotics in *E. faecalis*.

In order to further explore the genetic environment of antibiotic resistant genes in multidrug-resistant *E. faecalis*, we isolated a multidrug-resistant *E. faecalis* ESC1 from the feces of a cat in a pet hospital. The whole genome of *E. faecalis* ESC1 was sequenced. Bioinformatics analysis showed that the resistance of this strain to antibiotics was mainly mediated by the resistance genes carried by the mobile elements, and there was a risk of resistance transmission. In addition, we found a novel composite transposon carrying two aminoglycoside resistance genes *aac(6′)*-*aph(2″)*. Companion animals could be a potential threat and repository for carrying diseases and drug resistant bacteria to human health (Yuan et al., 2023), therefore, attention should be paid to the health of humans and companion animals in order to avoid its spread.

## 2. Materials and methods

### 2.1. Bacterial identification, conjugation and antimicrobial susceptibility testing

Samples of feces in this study were collected in 2018 from cats from a pet hospital located in the Jilin Province, China. A sample of 1 g of feces from each cat was inoculated into 2 mL phosphate buffer saline (PBS), and was directly streaked onto brain heart infusion (BHI) agar and incubated at 37°C overnight. According to Dong et al.’s (2019) study, 16S rRNA sequences of different forms of colonies were amplified using universal primers, 27F and 1492R. The primer sequence was as follow: F: 5′-AGAGTTTGATCCTGGCTCAG-3′, R: 5′-GGTTACCTTTACGATT-3′. The DNA of the isolated strains was extracted using the DNA extraction kit (Ezup cfDNA extraction kit, Sangon Biotech Co., Ltd., Shanghai, China). The amplified products were sequenced by Biotechnology Co., Ltd., Beijing, China. The sequencing results were compared using NCBI BLAST search. The isolated strains were tested for antibiotic sensitivity by micro broth dilution method.

According to the method of Vaser et al. (2017), a reverse PCR assay was established to detect the presence of circular intermediates. When linear transposon formed a circular intermediate between the two IS*1216E* elements, the product was amplified by PCR using reverse primers. The primer sequence was as follow: F: 5′-TCGGATTCAAGGACTCTTCAATCAAG-3′, R: 5′-AGAACTCTTATGTCCAATTCCACCACT-3′.

Conjugation transfer test was carried out according to the test method of Yu et al. (2022). To put it simply, rifampin-resistant *Escherichia coli* strain ATCC 25922 and rifampin-resistant *Staphylococcus aureus* ATCC 29212 were used as recipient strains. Bacteria were cultured in BHI broth medium at 37°C overnight, and then diluted at 1:100 in fresh broth to expand the culture to OD_600_ = 0.5. Bacterial precipitates were then collected by centrifugation and resuspended in BHI broth. The final conjugation system (total volume of 2 mL) consisted of 1 mL donor and 1 mL recipient bacteria. BHI agar supplemented with rifampicin (25 mg/mL) and tetracycline (50 mg/mL) was used for transconjugant selection after incubation at 37°C for 15 h.

Antimicrobial susceptibility testing of the *E. faecalis* ESC1 and its transconjugants was performed using broth microdilution method and the results were interpreted according to Clinical and Laboratory Standards Institute (CLSI) recommendations (Lubbers, 2023). A total of 9 antimicrobial agents tested were as follows: erythromycin, tetracycline, vancomycin, linezolid, ciprofloxacin, norfloxacin, moxifloxacin, chloramphenicol and florfenicol. *E. coli* ATCC 25922 and *S. aureus* ATCC 29212 served as a quality control strains.

### 2.2. Genome sequencing, genome assembly and bioinformatics

*E. faecalis* ESC1 genomic DNA was extracted using the FastPure Gel DNA Extraction Mini Kit (Vazyme, Nanjing, China) and quality checked for purity, concentration and integrity using Nanodrop 2000 (Thermo Scientific, MA, USA), Qubit 4 (Thermo Scientific, MA, USA) and 0.35% agarose gel electrophoresis in order to extract high quality genomic DNA. BluePippin automated nucleic acid recovery system (Sage Science, MA, USA) was used to recover large fragments of DNA. SQK-LSK109 ligation kit was used to construct the library. The genomic DNA was sequenced on the Oxford Nanopore platform (Jain et al., 2016; Wang et al., 2021). Assembly of filtered subreads was performed using Canu v1.5 software (Koren et al., 2017). The assembly results were corrected by Racon v3.4.3 software using third generation subreads (Vaser et al., 2017). Cyclisation and adjustment of start sites were performed by Circlator v1.5.5 software (Hunt et al., 2015). Further error correction was performed using second generation data using Pilon v1.22 software to obtain a more accurate genome for subsequent analysis (Walker et al., 2014).

Antimicrobial resistance genes were identified using the ResFinder 4.1.^1^ The mobile genetic elements were analyzed using the ISFinder,^2^ the Transposon Registry.^3^ Sequence analyses were conducted using the RAST^4^ and BLAST functions.^5^ For sequence comparisons, the BLAST algorithm^6^ was used. Using the assembled and predicted genome information, such as tRNA, rRNA, repeat sequence, GC content and gene function information, the genome circle map was drawn by Circos v0.66 software (Krzywinski et al., 2009).

## 3. Results

### 3.1. Minimum inhibitory concentration of *E. faecalis* ESC1 and transconjugants

A total of 11 cat fecal samples was collected from pet hospitals in Jilin province, China in 2018 and 28 strains were isolated. A strain of *E. faecalis* was identified by the 16S rRNA sequencing (CP118057.1: per.identity: 100%; e-value: 0; query cover: 100%) (Frank et al., 2008). The antimicrobial susceptibility phenotypes of *E. faecalis* ESC1 were determined using Muller–Hinton (MH) broth. The results showed that the strain was resistant to erythromycin, tetracycline, linezolid, ciprofloxacin, norfloxacin, moxifloxacin, chloramphenicol, florfenicol and sensitive to vancomycin (Table 1). As mentioned above, the conjugation experiments were conducted according to the international standard definitions. Minimum inhibitory concentrations (MICs) of different antimicrobial agents against *E. faecalis* ESC1 showed high levels of antibiotic resistance, representing multidrug-resistant strains. Transconjugants exhibited multidrug resistance. The MICs of *E. coli* and *S. aureus* to the tested antibiotics increased by 8–128 times and 2–256 times, respectively.

**TABLE 1:** MICs of different antibiotics against the *E. faecalis* ESC1 and its transconjugants.

μ g/mL										
	**GEN**	**ERY**	**TET**	**VA**	**LZD**	**CIP**	**NOR**	**MFX**	**CHL**	**FFC**
ESC1	256	128	128	1	8	256	16	32	64	128
ATCC 25922	0.5	–	2	–	–	0.125	2	0.125	8	8
ATCC 29212	16	0.25	2	1	4	1	2	0.25	4	8
ATCC 25922 transconjugants	64	–	128	–	–	0.125	2	0.125	128	64
ATCC 29212 transconjugants	256	128	64	1	8	1	4	0.25	128	64

GEN, gentamicin; ERY, erythromycin; TET, tetracycline; VA, vancomycin; LZD, linezolid; CIP, ciprofloxacin; NOR, norfloxacin; MFX, moxifloxacin; CHL, chloramphenicol; FFC, florfenicol.

### 3.2. Characterization of *E. faecalis* ESC1

**FIGURE 1:**
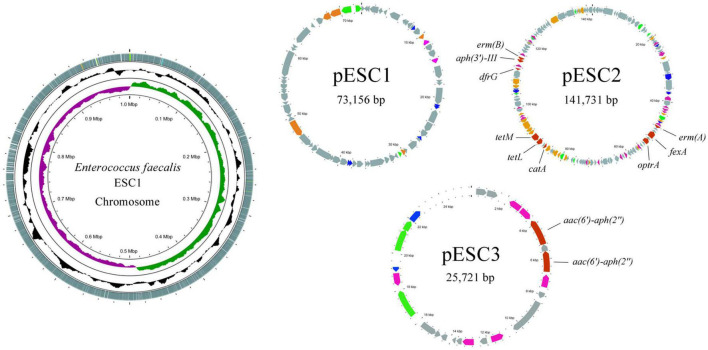
Circular representation of the *E. faecalis* ESC1 genome (chromosome and three plasmids). Moving inward in the chromosome circular map, slots 1–3 (slot 1, GC skew; slot 2, GC content; slot 3, open reading frames). Red for antibiotic resistance genes, orange for conjugation transfer of related genes, green for plasmid replication and maintenance related genes, gray for hypothetical proteins and pink for mobile genetic elements.

Based on the MIC study mentioned above, ESC1 was selected for genome-wide sequencing to clarify the potential mechanisms of multidrug resistance. A CIRCOS Circular representation of the ESC1 genome with annotated genes was constructed. The genome of *E. faecalis* ESC1 contained a chromosome and three plasmids (pESC1, pESC2, and pESC3) (Figure 1). The size of chromosome DNA of ESC1 was 2,887,950 bp and the ratio of guanine-cytosine content in the genome was 37.5, which is consistent to other reported *E. faecalis* genomes. The chromosomal DNA and plasmid sequences of *E. faecalis* ESC1 were deposited in NCBI GenBank with the accession numbers CP115992, CP115993, CP115994, and CP115995, respectively.

### 3.3. Antibiotic resistance genes in *E. faecalis* ESC1

Antibiotic resistance-associated genes were predicted according to ResFinder. ESC1 contained 11 resistance genes [*erm*(A), *erm*(B), *optr*A, *fex*A, *cat*A, *aph(3′)-III*, *aac(6′)*-*aph(2″)*, *tet*(M), *tet*(L), *dfr*(G)] and one resistance-related mutation, a serine at amino acid position 80 in the *parC* gene was mutated into leucine. These genes may confer resistance to macrolide, aminoglycoside, tetracycline, trimethoprim, oxazolidinone and phenicol.

### 3.4. Characterization of multidrug-resistant genomic elements

**FIGURE 2:**
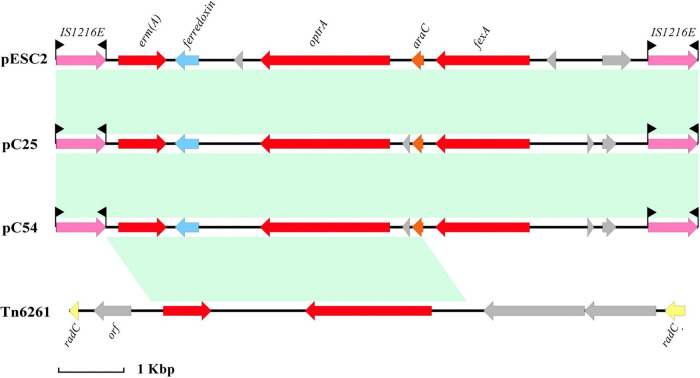
Organization of the composite transposon flanked by the insertion sequence IS*1216E* and comparison with related regions. The distance scale is given in kilobase. Genes are denoted by arrows. Genes, mobile elements and other features are colored based on their functional classification. Shading denotes regions of homology (nucleotide identity > 95%). The accession numbers of pC25, pC54, and Tn*6261* for reference are CP030043.1, CP030046.1, and KU354267.1, respectively.

**FIGURE 3:**
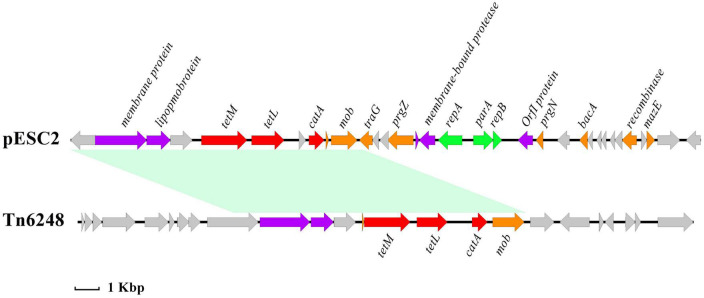
Linear illustration of the MDR region of pESC2 (69,630–96,200 bp) and comparative analysis of this region: Tn*6248* (accession number KP834592). The distance scale is given in kilobase. Red for antibiotic resistance genes, orange for conjugation transfer of related genes, green for plasmid replication and maintenance related genes, gray for hypothetical proteins and purple for other genes.

The size of plasmid pESC2 was 141,731 bp with 156 ORFs, and an average GC content of 34.4%, carrying 9 antibiotic resistance genes. Interestingly, plasmid pESC2 showed a very similar structure (99.99% nucleotide sequence identity and 69% query coverage) to plasmid pKUB3006-1 (Supplementary Figure 1), accession number AP018539.1. According to the observed similarities, these two plasmids may have evolved from each other or from the same ancestor. A part of a similar region contained 6 drug resistance genes. The *optr*A gene encoded an ATP binding cassette (ABC)-F protein, which was located on a composite transposon and flanked by the insertion sequence IS*1216E*. In addition, the composite transposon carried the antibiotic resistance genes *erm*(A) and *fex*A (Figure 2). According to the method of He et al. (2016), a reverse PCR assay was established to detect the presence of these circular intermediates between the two IS*1216E* elements in *E. faecalis* ESC1. The results of reverse PCR identification are shown in Supplementary Figure 2, We used the designed reaction PCR primers to amplify a target band at 2,618 bp, demonstrating the existence of a structure in which two IS*1626E* mobile primers are tandemly linked together. The results demonstrated the presence of the predicted circular intermediates, suggesting that this composite transposon could mediate the transfer of drug resistance genes. In addition, the genes *tet*(M), *tet*(L) and *cat*A, which mediate tetracycline and chloramphenicol resistance were located in a region similar to the Tn*6248* transposon (Figure 3).

**FIGURE 4:**
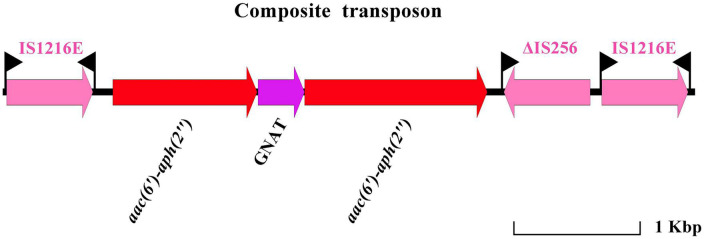
Linear illustration of the novel composite transposon of pESC3.

It is worth noting that a novel compound transposon located on plasmid pESC2 was found (Figure 4). The isotropic insertion sequence IS*1216* was located on both sides of the composite transposon. The gene encoding the GNAT family of acetyltransferases was located between two aminoglycoside resistance genes [*aac(6′)*-*aph(2″)*]. Downstream of one of the aminoglycoside resistance gene contained the remaining of an insertion sequence IS*256*. In contrast to the previously reported IS*256*, the upstream of this IS*256* transposase was truncated by IS*1216* or IS*256* carries an *aac(6′)*-*aph(2″)* gene inserted into the composite transposon. To the best of our knowledge, there are no reports of two *aac(6′)*-*aph(2″)* genes mediated by IS*1216* in tandem on a single composite transposon. When located in the same orientation, the recombination between two copies of IS*1216E* resulted in the generation of a loop containing the bracketed region plus one copy of the IS*1216E* element. Therefore, the novel composite transposon increased the risk of antibiotic-resistant gene dissemination. In addition, chromosomal DNA analysis of ESC1 revealed the presence of an integrative and conjugative element (ICE) region (143,268 bp in size with a guanine-cytosine content of 34.45%). The ICE was flanked by a 15-bp target site duplication (TAAATAATTGTTGGT) (Supplementary Figure 3).

### 3.5. Gene mapping

**FIGURE 5:**
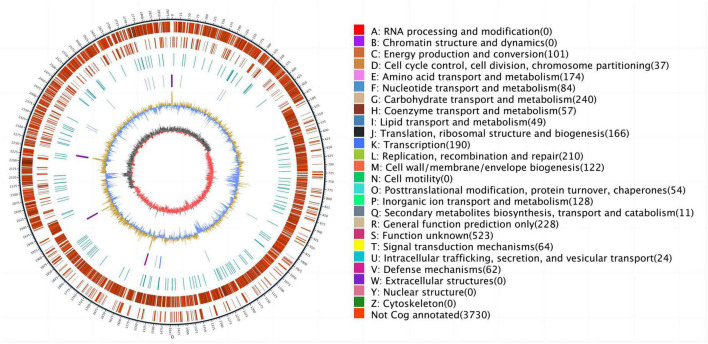
The outermost circle is marked with genome size, and each scale is 5 kb; the second circle and third circle are genes on the positive and negative chain of the genome, respectively, and different colors represent different functional classifications of COG; the fourth circle is a repetitive sequence; the fifth circle is tRNA and rRNA, blue is tRNA and purple is rRNA; the sixth circle is GC content, the light yellow part indicate that the GC content in this region is higher than the average GC content of the genome, the higher peak value, greater difference with the average GC content, and the blue part indicate that the GC content in this region is lower than the average GC content of the genome; the innermost ring is GC skew, dark gray represent the area where the G content is greater than C, and red represent the area where the C content is greater than G.

CIRCOS Circular genome visualization can more clearly explore the positional relationship of genome components in a genome. The loop diagram of assembled genome is shown in Figure 5. Except for the genes not annotated to function, we observed that 240 genes were implicated in carbohydrate transport and metabolism, 210 genes were implicated in replication, recombination and repair, 190 genes were implicated in transcription, 174 genes were implicated in amino acid transport and metabolism, 166 genes were implicated in translation, ribosomal structure and biology, 128 genes were implicated in inorganic ion transport and metabolism, 122 genes were implicated in cell wall/membrane/envelope biology and 101 genes were implicated in energy product on and conversion. These genes all have key functions in the bacterial growth.

## 4. Discussion

According to reports, *E. faecalis*, as an opportunistic pathogen, was mainly isolated from pig tissue and human tissue samples in the past (Getachew et al., 2013; Chen et al., 2020; Chilambi et al., 2021). As a companion animal, cats live with human beings, and even are regarded as family members among individuals, so they are very close to human life (Overgaauw et al., 2020). A data on fecal *E. faecalis* isolated from companion animals likes dogs and cats have showed that the isolation rate of *Enterococcus* was 60–80%, while most of these *Enterococcus* were multidrug-resistant strains. This suggests that companion dogs and cats may be carriers of anti-microbial drug resistance genes, with the potential of transfer to humans (Jackson et al., 2009). Multidrug-resistant strains are defined as having drug resistance to three types of antibiotics commonly used in the clinics (Magiorakos et al., 2012). We isolated a multidrug-resistant *E. faecalis* ESC1 from fecal samples of sick cats from pet hospitals, and found that it was resistant to nine antibiotics from five major tested groups and was therefore, termed as a multidrug-resistant strain (Magiorakos et al., 2012). In addition, the drug resistance of this strain can be horizontally transferred. In our experiment, the MIC of the tested antibiotics against recipient strains was significantly increased after co-culture with ESC1. We are not sure whether the plasmid can conjugate autonomously. We did not sequence and verify the plasmids in the recipient strains. This is the deficiency of our experiment. However, the antibiotic resistance phenotype of the transconjugant is consistent with the antibiotic resistance mediated by the antibiotic resistance gene on the plasmid. Moreover, this strain can promote the spread of antibiotic resistance genes and lead to the increase of antibiotic resistance of other strains. It can be seen that this aggravates the risk of the spread of drug resistance and becomes a potential threat to human health.

In order to investigate the cause of multidrug resistance in the *E. faecalis* ESC1, the whole genome was sequenced and bioinformatics analysis was carried out. We found the mutation of *parC* gene on the chromosome, which resulted in the resistance to fluoroquinolones. In addition, no resistance genes were found on the chromosome, rather resistance genes were mainly carried by movable plasmid. Therefore, this can explain the multidrug resistance phenotype and drug resistance spread of the strain. The strain carried three plasmids. No drug resistance genes were found in plasmid pESC1. Some regions of the pESC2 plasmid showed an organization highly similar to the pKUB3006-1 plasmid (99% nucleotide sequence identity with a query coverage of 69%). Based on the observed similarities, the two plasmids may have evolved from each other or from the same ancestor. Compared to the pKUB3006-1 plasmid, the pESC2 plasmid retained three resistance genes, *erm*(B), *aph(3′)-III* and *dfr*G, which conferred resistance to macrolides, aminoglycosides and trimethoprim. The *ant* (Mackenzie and Jeggo, 2019)*-Ia* and *lnu(B)* genes were missing. This might be a loss in the course of evolution. Apart from a region similar to the pKUB3006-1 plasmid, a 56.2 kb sized region located on pESC2 carried six drug resistance genes. The *optr*A, *fex*A and *erm*(A) genes were located on a composite transposon flanked by an insertion sequence IS*1216E*. Similar to the structure of the pE419 plasmid reported by He et al. (2016), this transposon carries the *optr*A, *fex*A and *erm*(A) genes with a reverse insertion sequence. By comparing with NCBI database, we found other similar structures as well. These similar structures were located on plasmid pC25(CP030043.1), pC54(CP030046.1), and transposon Tn*6261*(KU354267.1), respectively. Gene *optr*A encodes an ABC-F protein, which mediates the drug resistance to oxazolidinone antibiotics (Almeida et al., 2020). This poses a severe treatment challenge to human and veterinary medicine. In this study, we found that this region of Tn*6248* carried three resistance genes, *tet*(M), *tet*(L) and *cat*A genes, while the transposon also retained genes encoding plasmid recombination, binding, lipoprotein and membrane protein. All this suggests that the drug resistance of this transposon can be still spread with the transfer of the plasmid. Analysis showed that the plasmid underwent extensive homologous recombination, resulted in the acquisition of these resistance genes. In addition, we identified many mobile elements and mosaic structures. The above analysis shows that multiple recombination events may have been experienced on plasmid pESC2, resulting in these drug resistance genes. By analyzing the genomic content, we found that the mobile elements carrying antibiotic resistance genes are active among bacteria. This is an important reason for the wide spread of antibiotic resistance genes. Irrational use of antibiotics is an important factor to promote the spread of antibiotic-resistant genes. Therefore, we once again recommend rational use of antibiotics to slow down the spread of bacterial resistance.

Severe enterococcal infections are usually treated using a combination of a cell-wall-active agent (β-lactam or vancomycin) and aminoglycoside (typically gentamicin) (Zhang et al., 2018). The first clinical report of HLGR *Enterococcus* was completed in France in 1979, and it was widely reported afterward (Horodniceanu et al., 1979; Qu et al., 2006; Dadfarma et al., 2013; Liu et al., 2013; Jannati et al., 2020). HLGR is mainly mediated by the *aac(6′)*-*aph(2″)* gene encoding the bifunctional enzyme 6-acetyltransferase-2-phosphotransferase. The *aac(6′)*-*aph(2″)* gene is carried by plasmids in most cases and is located on the Tn*5281*-like transposon in *Enterococcus*. Zhang et al. (2018) have shown that *aac(6′)*-*aph(2″)* is carried and transferred by IS*256*. In our study, we identified a novel composite transposon on pESC3. Two *aac(6′)*-*aph(2″)* genes were tandemly linked to a transposon flanked by isotropic IS*1216*. To the best of our knowledge, there are no reports of two *aac(6′)*-*aph(2″)* genes mediated by IS*1216* in tandem on a single composite transposon. When located in the same orientation, the recombination between two copies of IS*1216E* resulted in the generation of a loop containing the bracketed region plus one copy of the IS*1216E* element (He et al., 2016). Therefore, the novel composite transposon increased the risk of antibiotic-resistant gene dissemination. ICE, which is located on chromosome, does not carry drug resistance genes, but it lays a hidden danger for the spread of drug resistance genes (Delavat et al., 2017). Genome-wide analysis of *E. faecalis* ESC1 has provided clarity on the reason for its multidrug resistance and the potential for transmission of resistance genes. However, the exact extent of the contribution played in the development of drug resistance in pathogenic bacteria remains unclear.

In general, we found that *E. faecalis* with multi-drug resistance is host to a large number of drug-resistant genes, which may pose a threat to public health and safety and requires great attention. The characteristics of genetic evolution of *E. faecalis* will provide a reference for preventing zoonosis caused by *E. faecalis*.

## Data availability statement

The datasets presented in this study can be found in online repositories. The names of the repository/repositories and accession number(s) can be found below: https://www.ncbi.nlm.nih.gov/, CP115992, CP115993, CP115994, and CP115995.

## Author contributions

L-CK and H-XM contributed to the conception of the study. X-SL, YQ, Y-zL, and J-zX contributed significantly to the analyses conducted in the study and the preparation of the manuscript. X-SL, IM, P-hL, and X-yL performed the data analyses and wrote the manuscript. YM and D-mZ helped perform the analyses with constructive discussions. All authors contributed to the article and approved the final version.

## Footnotes

1
https://cge.food.dtu.dk/services/resfinder

2
https://www-is.biotoul.fr/

3
https://transposon.lstmed.ac.uk/

4
https://rast.nmpdr.org/

5
http://blast.ncbi.nlm.nih.gov/blast.cgi

6
www.ncbi.nlm.nih.gov/blast
