# Supplementary material for: Genetic characterization of MDR genomic elements carrying two aac(6′)-aph(2″) genes in feline-derived clinical Enterococcus faecalis isolate
Source: Front Microbiol. 2023 Jul 27;14:1191837. doi: 10.3389/fmicb.2023.1191837 (PMC10413266; doi:10.3389/fmicb.2023.1191837)
Supplement: Supplementary file 1 [file Data_Sheet_1.docx]

**Title:** Genetic characterization of MDR genomic elements carrying two aac(6')-aph(2'') genes in feline-derived clinical Enterococcus faecalis isolate

Xue-Song Li^1,2†^, Yu Qi^1,2†^, Jun-ze Xue^1,2^, Peng-hui Li^1,2^, Xuan-yu Li^1,2^, Inam Muhammad^1,2,3^, Ya-zhuo Li^1,2^, Dao-mi Zhu^1,2^, Ying Ma^4^, Ling-Cong Kong^1,2*^and Hong-Xia Ma^1,2,5*^

^1^ Department of Veterinary Medicine, College of Animal Science and Technology, Jilin Agricultural University, Xincheng Street No. 2888, Changchun 130118, China.

^2^ The Key Laboratory of New Veterinary Drug Research and Development of Jilin Province, Jilin Agricultural University; Xincheng Street No. 2888, Changchun 130118, China.

^3^ Department of Zoology, Shaheed Benazir Bhutto University Sheringal, KPK, Pakistan.

^4^ Liaoyuan Animal Disease Prevention and Control Center, Renmin Street No.1006, Liaoyuan136200, China.

^5^ The Engineering Research Center of Bioreactor and Drug Development, Ministry of Education, Jilin Agricultural University; Xincheng Street No. 2888, Changchun 130118, China.

**^†^** These authors contributed equally to this work and share first authorship

**First authorship:**

1. Xue-song Li

College of Animal Science and Technology, Jilin Agricultural University, Xincheng Street No.2888, Changchun 130118, China. E-mail address: 20201587@mails.jlau.edu.cn

1. Yu Qi

College of Animal Science and Technology, Jilin Agricultural University, Xincheng Street No.2888, Changchun 130118, China. E-mail: 20200315@mails.jlau.edu.cn

^*^ Corresponding authors:
**^1.^** Ling-Cong KONG

College of Animal Medicine, Jilin Agricultural University; Xincheng Street No. 2888, Changchun 130118, China.

E-mail: lingcong@jlau.edu.cn

1. Hong-Xia MA

College of Animal Medicine, Jilin Agricultural University; Xincheng Street No. 2888, Changchun 130118, China. E-mail: hongxia0731001@163.com

**
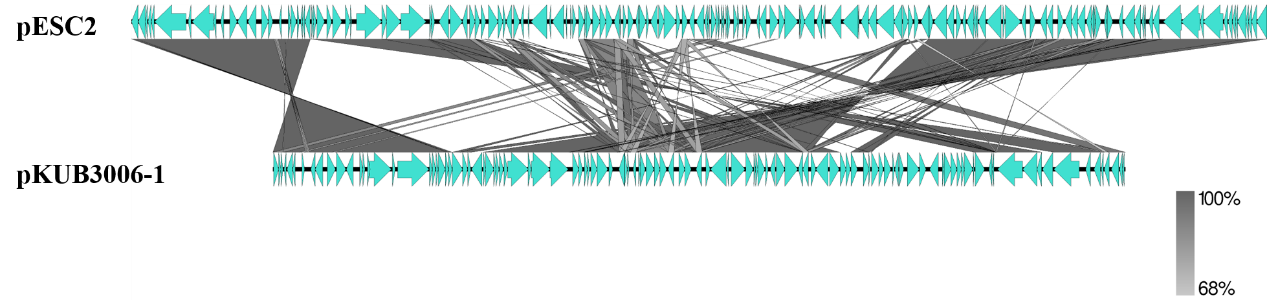
**

**Supplementary Fig. S1. Comparison of the genetic contexts pESC1 plasmid found in this study with pKUB3006 plasmid.** Sequence comparisons and map generation were performed using BLAST and Easyfig (version 2.1).

**
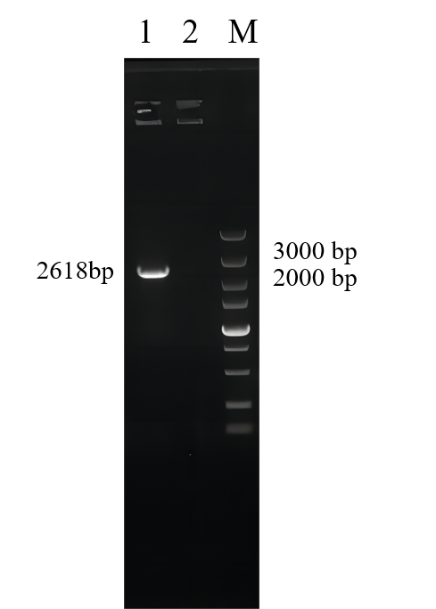
**

**Supplementary Fig. S2. Reverse PCR identifies ESC1 strain containing two IS*1216E* fragments.** M: DL 2 000 Marker, 1, Reverse PCR products, 2, Negative control.


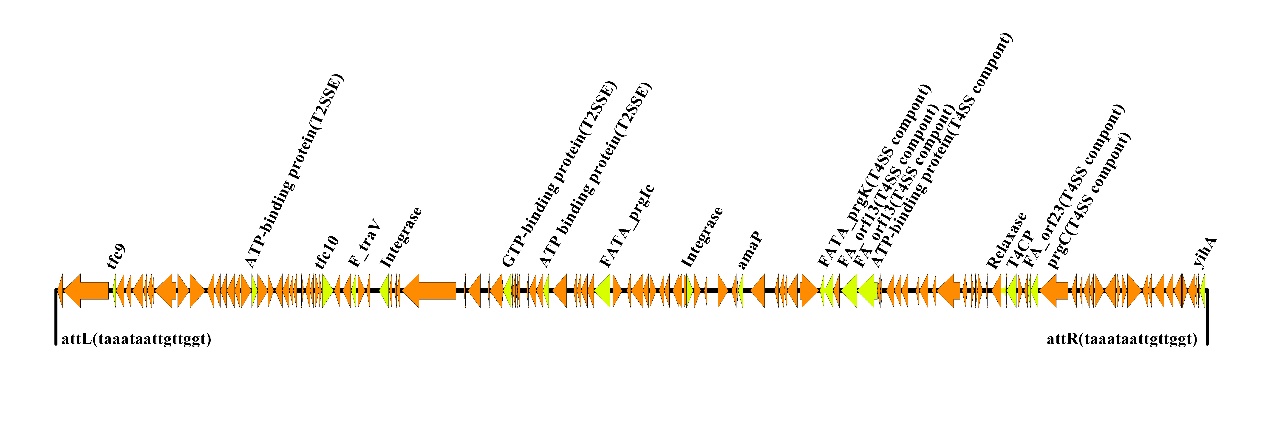


**Supplementary Fig. S3. Pattern diagram of the integrative and conjugative element (ICE) located on chromosome of ESC1 strain.** The yellow arrows represent the main genes of the ICE.
